# A randomized pragmatic care trial on endovascular acute stroke interventions (EASI): criticisms, responses, and ethics of integrating research and clinical care

**DOI:** 10.1186/s13063-018-2870-6

**Published:** 2018-09-19

**Authors:** Robert Fahed, Stefanos Finitsis, Naim Khoury, Yan Deschaintre, Nicole Daneault, Laura Gioia, Gregory Jacquin, Céline Odier, Alexande Y. Poppe, Alain Weill, Daniel Roy, Tim E. Darsaut, Thanh N. Nguyen, Jean Raymond

**Affiliations:** 1 Department of Radiology, Service of Neuroradiology, Centre Hospitalier de l’Université de Montréal (CHUM), University of Montreal, D03.5462B, 1000 Saint-Denis, Montreal, Quebec H2X 0C1 Canada; 2 Department of Interventional Neuroradiology, Rothschild Foundation Hospital, Paris, France; 3 Neurovascular Team, Division of Neurology, Department of Medicine, Centre hospitalier de l’Université de Montréal (CHUM), University of Montreal, Montreal, Québec Canada; 4 Department of Surgery, Division of Neurosurgery, University of Alberta hospital, Mackenzie Health Sciences Center, Edmonton, AB Canada; 5 Department of Neurology, Neurosurgery, and Radiology, Boston Medical Center, Boston, MA USA

**Keywords:** Thrombectomy, Stroke, Trial, Methodology, Ethics

## Abstract

**Background:**

The Endovascular Acute Stroke Intervention (EASI) trial was conceived as a pragmatic care trial, designed to integrate trial methods with clinical practice. Reporting the EASI experience was met with objections and criticisms during peer review concerning both scientific and ethical issues. Our goal is to discuss these criticisms in order to promote the pragmatic approach of care trials in outcome-based medical care.

**Methods:**

The comments and criticisms of 11 reviewers from 5 journals were collected and analyzed. The EASI protocol was also compared to the protocols of seven thrombectomy trials using the pragmatic-explanatory continuum indicator summary (PRECIS).

**Results:**

Main criticisms of EASI concerned selection criteria that were judged to be too vague and too inclusive, brain and vascular imaging methods that were not sufficiently prescribed by protocol, lack of blinding of outcome assessment, and lack of power. EASI was at the pragmatic end of the spectrum of thrombectomy trials.

**Conclusion:**

The pragmatic care trial methodology is not currently well-established. More work needs to be done to integrate scientific methods and ethical care in the best medical interest of current patients.

## Background

Clinical research and care are commonly conceived as distinct activities that must be separated. This conception encourages the conduct of clinical trials on the model of preclinical studies, where patients are used in experiments designed to gain knowledge for the potential benefit of future patients or society, but the results of such explanatory trials are often poorly applicable in practice [1, 2]. The received view misses the role research methods can play in guiding care under uncertainty, in the best medical interest of current patients. The publication of multiple trials on thrombectomy in acute stroke may serve to illustrate and discuss this problem.

The Endovascular Acute Stroke Intervention (EASI) trial is a care trial designed to offer thrombectomy to patients referred for acute stroke treatment [3]. At the time the trial was conceived, three previous trials had failed to show clinical benefit from endovascular treatment [4–6]. We were unwilling to accept those trials as definitive. This was partly because we felt our ability to recanalize intracranial vessels had substantially improved with the latest generation of endovascular devices [7], which had not properly been tested in these trials. Thus, we did not stop offering thrombectomy to many of our patients with severe strokes. Akin to many endovascular centers, and in the absence of randomized evidence for almost all neuro-interventions, we had been offering intra-arterial (IA) treatments for stroke for decades on a case-by-case basis. The main justification to attempt an IA intervention was on compassionate grounds: some patients were referred with such severe strokes that they were unlikely to survive without a debilitating deficit if nothing was done. We also felt that the techniques required for mechanical thrombectomy were similar to interventions we routinely performed, and we knew that the procedure could work (at least occasionally) if carried out rapidly enough from stroke onset. We did realize the dangers of practicing interventions without concomitantly verifying whether we were doing good or harm. We also recognized that reorganization of healthcare systems to ensure timely transfer of patients with acute stroke to thrombectomy centers without proof that it was beneficial would be impossible. Following our participation in IMS-3 [4], it was our intent to participate in one of the large-scale thrombectomy trials that were being planned, but there were concerns: many trials were by design deliberate attempts to show that thrombectomy could work in the best possible clinical circumstances [8, 9]. This is the approach of explanatory trials, clinical experiments using scientific protocols to test if therapy *can* work, or to discover mechanisms of action, while we needed a pragmatic design, because our primary objective was to offer a promising yet unvalidated intervention to patients in need of urgent care, and at the same time we would evaluate in real time if our innovative treatment would improve outcomes in the reality of everyday practice [2].

We were at the time working out the details of how to design care trials [10], elaborated in the spirit of a “learning health care system”, in which “knowledge generation is so embedded into the core of the practice of medicine that it is a natural outgrowth … leading to continual improvement in care.” [11] A number of care trials we have designed are ongoing [12–15]. Integrating trials within medical practice requires adjustments on both the research and care fronts: on the one hand the trial design is adapted to offer care in the best medical interest of each patient; on the other hand, clinical practice is disciplined to acknowledge the current uncertainty and act accordingly. Unsurprisingly, care trials have raised concerns from both sides: the design adapted to care has been criticized by conventional trialists, and the randomized allocation that protects patients from unvalidated interventions remains poorly accepted by the clinical community.

Many conventional trial protocols seem to put the patient at the service of the research question, sometimes to the detriment of individual patient care. A care trial design is meant to address the hurdle of improving patient outcomes with real-time verification. Integrating trial participation in the care setting requires that each item of the study protocol is in agreement with the central principle of care ethics: is this aspect of the trial design in the best medical interest of the patient? [10, 16–18].

EASI was one of the first studies carried out using care-trial methodology [3, 10]. Launched in January 2013 after publication of three negative endovascular stroke trials, EASI was a randomized trial comparing the clinical outcome (modified Rankin score (mRS) at 3 months) of patients presenting with severe ischemic stroke treated with standard care plus mechanical thrombectomy versus standard care alone within 5 h of symptom onset or beyond this time point if clinical–imaging mismatch was observed (ClinicalTrials.gov: NCT02157532). We believed that thrombectomy could be beneficial, but in the absence of randomized evidence, it could also be useless or even harmful. Thus, we elected to continue offering thrombectomy, but only within a care-trial context. The EASI protocol met all the characteristics of a care trial: wide selection criteria designed to include all patients being referred for thrombectomy, a 1:1 randomized allocation ratio with no extra risks, no extra tests, no extra costs, and simple case report forms to be completed by normal care personnel. Technical details regarding the interventional procedure and other aspects of care, including follow-up visits and outcome assessment were to be performed according to local practices by everyday practitioners.

EASI was interrupted when the results of MR CLEAN, the first trial showing the benefits of thrombectomy became public in October 2014 [19]; at that point 77 (of 480 planned) EASI patients had been recruited at a single center. Other thrombectomy trials that were ongoing in different countries were also prematurely interrupted; results were analyzed and published in major journals. We decided then to publish the EASI results up to that date (even though no significant differences were shown) [3] in order to share our experience and in the hope of recruiting more participating centers for the continuation of the care-trial effort (after design modifications to address the remaining uncertainties in IA stroke treatment). The manuscript was turned down by 5 journals, based on the criticisms of 11 reviewers, before it was finally accepted for publication [3]. The purpose of this paper is to respond to the most frequent criticisms that were made during the peer review process, and to discuss how and why care trials differ in design and conduct from explanatory trials.

## Methods

### Objectives

To compare and explain how care trials differ in design and conduct from conventional trials and to review and address the most frequent criticisms that were made during the peer review process for the EASI trial.

### Methods

Two authors (RF and JR) reviewed the collected comments from the 11 reviewers and 5 editors of the 5 journals to which EASI was submitted, grouped them by similarity of reviewer’s concern, and sorted them by frequency of occurrence. They were further classified as either “scientific concerns” (concerns regarding trial design which reviewers judged as scientific weaknesses) or “ethical concerns” (concerns addressing namely the ethics of the design and conduct of the trial). Criticisms based on erroneous reading or misinterpretation of the manuscript (such as criticizing the severe imbalances in the number of patients who were Tissue Plasminogen Activator (tPA) eligible - while there were 23 patients in each group) were not retained.

The protocols of the seven thrombectomy trials that were registered in ClinicalTrials.gov and Current Controlled Trials and published prior to February 2017 [8, 9, 19–23] were also reviewed in consensus agreement by three authors (RF, SF, and DR) for an item-by-item comparison with the EASI protocol, using the diagrammatic tool of the pragmatic-explanatory continuum indicator summary (PRECIS)-2 framework [24, 25]. PRECIS is a tool to help situate clinical trials on the continuum from explanatory (idealized conditions) to pragmatic (usual circumstances) [24]. The second version of this tool (PRECIS-2) evaluates nine domains of trial design (eligibility, recruitment, setting, organization, flexibility (delivery), flexibility (adherence), follow up, primary outcome, and primary analysis): each characteristic is scored from 1 (very explanatory) to 5 (very pragmatic) [25]. The item “flexibility (adherence)” (i.e. “what measures are in place to make sure participants adhere to the intervention?”) was judged to be not applicable to the domain of thrombectomy, and was not evaluated in this study.

## Results

**Fig. 1:**
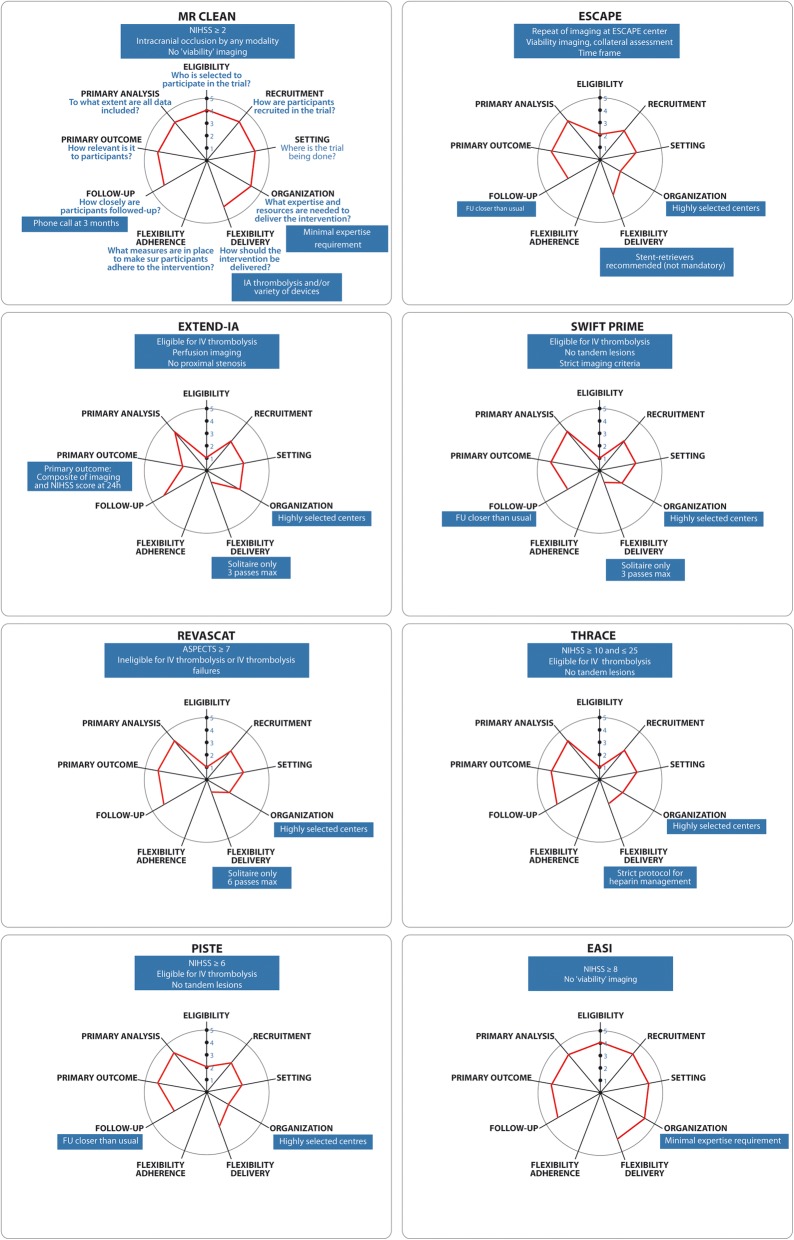
Assessment of all the protocols of trials of mechanical thrombectomy published between 2015 and 2017, using the pragmatic-explanatory continuum indicator summary (PRECIS) diagram tool. MR CLEAN and EASI were the most pragmatic trials, according to the PRECIS. Please notice that no trials other than MR CLEAN and EASI scored more than 2 points on the “Eligibility” spoke, due to their narrow selection criteria, leading to poor generalizability to clinical practice

Most criticisms from peer reviewers concerned the pragmatic choices of the EASI trial design, at the pragmatic end of the spectrum of recently published thrombectomy trials. The PRECIS-2 diagrams of the various thrombectomy trials [8, 9, 19–23], including EASI [3], are shown for visual comparison in Fig. 1.

Most trials were by design near the explanatory end of the spectrum: they restricted eligibility to a narrow class of patients susceptible to show therapy can work in optimal circumstances; some trials restricted participation to centers of excellence; details of thrombectomy techniques and devices were rigidly imposed in other protocols; some required repeating imaging studies without clinical indications to do so while this would delay therapy; some trials used outcome measures that were not clinical but mechanistic (Fig. 1). By comparison, EASI was with MR CLEAN at the pragmatic end of the spectrum, with none of the aforementioned restrictions.

EASI was offered to all patients referred for endovascular treatment of acute ischemic stroke. Inclusion criteria were broad: age ≥ 18 years, National Institute of Health (NIH) Stroke Scale (NIHSS) ≥ 8, onset of symptoms within ≤ 5 h or the presence of clinical–imaging mismatch, and suspected or proven occlusion of the M1 or M2 segments of the middle cerebral artery (MCA), supraclinoid internal carotid artery (ICA), or basilar artery. Vascular imaging was not mandated in the protocol. The exclusion criteria were established infarction or hemorrhagic transformation of the target symptomatic territory and co-morbidities associated with a poor 90-day outcome.

All trials excluded some patients per protocol, and thus no trial was attributed a score of 5/5 for patient selection. EASI and MR CLEAN were both given a score of 4/5, despite differences in eligibility criteria MR CLEAN was most inclusive in terms of neurological deficits (NIHSS > 2), but excluded patients who could not be treated within 6 h, as well as patients with posterior circulation strokes. The latter were included in EASI, as well as patients beyond 5 h if a clinical–imaging mismatch was judged to be present, whereas patients with minor neurological deficits (NIHSS < 8) were excluded. Other differences between the last two trials were mandatory vascular imaging and blinded assessment of the primary outcome in MR CLEAN. EASI was meant to be multicentric, but only one center was active at the time of trial interruption, while MR CLEAN was a national multicenter study (reimbursement for thrombectomy in The Netherlands was conditional on participation).

The seven most frequent criticisms regarding EASI are listed in Table 1. Scientific concerns: several criticisms concerned the selection of patients. Reviewers (six reviewers/three journals) disapproved that EASI did not require non-invasive vascular imaging to prove proximal vessel occlusion, claiming it was “mandatory in the post IMS-3 era” and “standard in all other trials”. EASI’s brain imaging selection criteria were qualified as “too vague” or “too broad” (six reviewers/four journals). Many reviewers (six reviewers/three journals) would have preferred to see a homogenous population of similar patients, which excluded posterior circulation occlusions (three reviewers/two journals). Additional criticisms stated that EASI was underpowered due to premature trial interruption (four reviewers/two journals) and that the results were inferior to those of other thrombectomy trials (five reviewers /two journals). The lack of blinding of assessors of the primary outcome (functional status 3 months) was also criticized (four reviewers/three journals).

**Table 1:** The seven most frequent criticisms regarding the EASI trial

Criticism	Number of journals	Number of reviewers	Examples of reviewers' comments
Early interruption resulting in an underpowered trial	2	4	“The timing of the release of the data is unfortunate since the majority of high quality trials in this area have now been published” “The numbers are too small to permit any meaningful interpretation, especially having recruited only 16% of the sample size predicted to be required”
Results inferior to other RCTs	2	5	“The rigorous application of well-validated and rigidly implemented imaging selection criteria in EXTEND-IA are a lesson in the importance of methodological standards, achieving a clear result in a smaller number of subjects than included in the present study” “The authors suggest that they have a more virtuous approach than more rigorous trialists who exclude patients who do not meet inclusion and exclusion criteria that may go beyond standard care: their results speak for themselves. Outcomes were not improved, mortality was higher, there were more SAEs in the IAT group […]”
Lack of blinding for the mRS at 3 months	3	4	“Lack of outcome blinding using mRS is a major flaw” “Lack of blinding may preclude its use in some meta- analyses where this is a minimum prerequisite”
No vascular imaging for patient selection	3	6	“The main distinguishing feature of the present study is the lack of vascular imaging as a qualifying criterion, something adopted post-IMS-3 by all endovascular trials” “The confirmation of large artery occlusion is not mandated in this design which is also another mandatory criteria”
Brain imaging selection criteria “too vague”	4	6	“The selection criteria are very vague which is probably the point of a care trial”
Population too heterogeneous. Inclusion of posterior-circulation strokes	3	6	“While some had similar small sample sizes, also because of early termination, most showed a strongly positive result due to more rigorous patient selection” “The wisdom of mixing anterior circulation cases with basilar artery occlusion patients is highly questionable, since the outcomes are very different” “The authors consider their design as “pragmatic care trial” with wide inclusion criteria. This might be a good idea once a method has shown its effect in a core publication. Such design could kill a therapy without giving in further chances for proving its worth.”
Ethical concerns. Patients “wasted” in a small trial.	2	4	“What was the justification to perform […] a mono-center trial which would have never been able to recruit 480 patients in a time frame before the 5 ongoing studies would have been terminated?” “The study recruited patients in a single center in parallel to other studies, e.g. THRACE and SWIFT-PRIME. Their patients are “wasted” for a small study of limited validity with “parsimonious” data collection […] instead of enrolling the patients into larger trial efforts.”

*mRS* modified Rankin Scale, *RCT* randomized controlled trial, *SAE* severe adverse event

### Ethical concerns

Finally, reviewers had ethical concerns (four reviewers/two journals); for example, one reviewer was concerned about the ‘wasted’ contribution of research participants: “Instead of enrolling the patients into larger trial efforts, their patients are ‘wasted’ for a small study of limited validity.” “The result of this approach was an underpowered trial which does not really contribute to our approach to thrombectomy.”

## Discussion

Care trials are at the pragmatic end of the explanatory–pragmatic continuum [10]. Explanatory trials are clinical experiments best adapted to explore causality at a fundamental level. Their design resembles the methods of the laboratory, where an experiment is an artificial set-up at the service of empirically verifying a theory, capable of isolating a mechanism or of revealing a phenomenon that would be difficult to observe if conditions were natural. The meticulous control of the experimental setting, the fixed and fastidious protocols, the close monitoring, and the careful selection of a homogenous group of subjects best suited to maximize the difference in outcomes between the experimental and the control groups, all parts of the explanatory design aim at revealing with sensitivity any signal that a new treatment or mechanism has an effect. On the logical side, when an explanatory trial fails to show a signal with a sufficiently narrow confidence interval, one can safely conclude that treatment has no effect. However, when the outcome is positive, the results obtained in such a narrow subset of individuals in such artificial conditions can only rarely be generalized to clinical practice. In contrast, in pragmatic trials, the “experimental set-up” is already given: it is the real world of practice, where care is normally provided. Because pragmatic trials aim at assessing the comparative benefits of two or more management options as they are practiced in normal conditions in all patients in need of care, their results are more readily applicable in the real world. Both attempt to respect scientific norms, but they differ in the object and aims of the study: the explanatory design is fundamentally at the service of the treatment (it tries to answer the question: can it work in ideal circumstances?) The pragmatic design is at the service of medical practice (does treatment work under usual circumstances?). The aforementioned distinction is commonly said to be inappropriately bipolar, as most trial designs are somewhere along a continuum, and that the proper design of any given trial depends on the research question [24–26]. This is where care trials go one step further: while conventional trials, in a sense, use patients to answer a research question (and this is the source of most research ethics conundrums), in care trials research methods are at the service of patients [10]. The distinction between explanatory and pragmatic trials was recognized by Schwartz and Lelouch in 1967 [2]. They emphasized that “In the first place, fundamental research aimed at the identification of a biological hypothesis is done on a relatively arbitrary population which is ultimately treated as a means rather than an end; as such, the use of human subjects must be impermissible except in special cases. Normally, explanatory work must be done on animals, therapeutic trials on human subjects being limited to pragmatic experiments.” The distinction between explanatory and pragmatic trials and their diverging aims seem to be poorly recognized in our clinical neurovascular community, since many of the reviewers’ comments suggest that they would have preferred a less pragmatic design than the one proposed in EASI, as discussed below.

The PRECIS score is higher for pragmatic trials. EASI, along with MR CLEAN, was at the pragmatic end of the PRECIS score, as compared to other thrombectomy trials. The PRECIS diagram provides rapid and useful summative assessment of the degree to which a trial is considered pragmatic; nevertheless, it has several limitations including the inability to appropriately compare the incommensurable differences between trials (such as comparing NIHSS score limitations and selection by thrombus location).

In our view, the critiques concerning incomplete or poor clinical results were inappropriate, as EASI was still in progress when a more definitive pragmatic trial (MR CLEAN) was reported. Even though no claims regarding the primary trial hypothesis should be made based on underpowered trials, all trials should be reported transparently irrespective of the results, as recommended by the World Health Organization [27]. Other thrombectomy trials were prematurely interrupted as well at the time the results of MR CLEAN were communicated [8, 9, 20, 22, 23]. It is logical and ethical to interrupt a study in the presence of a significant advance in knowledge elucidated by the results of other trials. It is, however, difficult to understand why the publication of the results should be reserved for trials that reach a statistical verdict in favor of therapy. The main objective of the publication of the EASI results was to share and make public the experience of practicing a yet-to-be proven neuro-intervention within the context of a care trial. The procedure that should eventually be followed when an all-inclusive care trial is interrupted because there is a clear benefit (or harm) for a particular subgroup of patients has yet to be elucidated in detail. Normally, conditions for trial interruption and stopping rules are pre-specified by protocol. Priority is given, however, to providing a care research context for all other patients for whom no one knows what to do.

Blinding treatment allocation from whomever assesses the primary outcome is a widely recognized mean to decrease potential biases. But blinding is commonly considered a hallmark of research. While it is certainly compatible with a care trial [10], it remains foreign to clinical care as understood by our Ethics Committees. To integrate the trial into the routine care of patients in spite of the received views regarding the regulation of research, we had to zealously eliminate any items of the protocol identified as research (understood as “for the purpose of gaining generalizable knowledge”), and leave determination of the primary outcome to clinicians that had access to all the available information about the patient, to claim that “optimal care, in that context of uncertainty, was the trial”. In the future, blinded clinical adjudicators may become more widely accepted, as quality-of-care studies already introduce a third party in the evaluation of care. Blinding may also assure equal follow-up care.

The frequent criticisms of the reviewers regarding selection of patients reveals a lack of understanding of the aims of pragmatic trials. We will discuss first the role of brain imaging, as it illustrates the diverging aims of care trials, in which the medical interests of the patient come first, and the aims of explanatory trials, where priority is given to the demonstration of a treatment effect [10].

Some form of brain imaging prior to mechanical thrombectomy is normally required, at least to rule out hemorrhage. In EASI, patients could be included outside the 5-h time frame if “clinical–imaging mismatch” was judged to exist; in addition, patients presenting in any time window with “established infarction of the target symptomatic territory” were excluded from trial participation [3]. These criteria were intentionally created to be broad: unless the intervention could salvage presumably non-infarcted tissue at risk, there would be no point in recanalizing a vessel. Radiographic inclusion criteria were loosely defined in order not to rigidly impose sophisticated and time-consuming imaging examinations by protocol, and thus cause unnecessary research-related delays potentially detrimental to patient outcomes. Although advanced imaging was not imposed by protocol, it was not prohibited either, in a way to allow its use on a case-by-case basis if judged essential to clinical care by the treating physician. If advanced imaging techniques have the “potential to enrich the patient population … and deliver positive evidence with the minimum sample size” as claimed in some thrombectomy protocols [8], their use also has had the unwanted consequences of excluding patients that might have benefitted from mechanical thrombectomy. Despite recent randomized evidence that advanced imaging can identify patients who can benefit from thrombectomy in the extended window (> 6 h) [28, 29], there is lack of scientific data showing that advanced imaging can reliably identify patients who will *not* benefit from the intervention. Strict imaging criteria were not included in the protocols of other trials with the aim of improving individual patient outcomes; they were rather admittedly imposed to maximize the efficiency of the trial and to *sieve* patients for the retention of those who had a maximal chance of showing the treatment *could* work in optimal circumstances. Since they also had the potential to unnecessarily delay treatments in selected patients, these imaging tests cannot be imposed on all patients in a care-trial protocol. It is worth remembering that diagnostic tests must be rigorously assessed prior to serving as a diagnostic tool. The inter-observer reliability of the most commonly used early computed tomography (CT) changes grading scale (ASPECTS) that served to define the threshold for including patients in many research protocols has been shown to be insufficient for making the decision of whether or not to treat patients [30]. The value of any imaging test used for patients’ selection must also be verified, by first proceeding with randomized allocation of treatment options regardless of test results, to prove that the test can actually differentiate patients who respond from patients who will not respond to the treatment. None of the selection criteria or diagnostic tests used in past thrombectomy trials (including the more recent trials on the role perfusion imaging in treating patients presenting beyond 6 h) have so far been validated as necessary to improve patient outcomes.

Another hallmark of pragmatic trials (including care trials) is the flexibility in the delivery of individualized care. One crucial difference between routine practice, which is in essence individualized care, and a conventional research protocol, is that the latter usually requires clearly defined, standardized procedures which are implemented for each and every study subject. When a test is mandated by a research protocol, it is imposed on all patients, even those who may not clinically require it. In certain trials, this may only minimally affect care, but this is certainly not the case for a time-sensitive illness such as acute stroke. With the interests of patients placed first, care trials are fundamentally different: care trial protocols do not impose tests on patients that have not been proven to be of benefit to the patient. Accordingly, non-invasive vascular imaging was not enforced in EASI, even though 80% of EASI patients had CT angiography (CTA)-proven proximal occlusions. In fact, the criticism about the absence of mandated CTA imaging prior to trial inclusion only applied to 15/77 (19%) patients. Negative catheter angiography (no occlusion found at time of intervention) occurred in only 3/77 (4%) of patients. It is worth noting here that the reviewers’ concerns did not revolve around the unnecessary risk of catheter angiography; their concern was primarily regarding the risk of including patients that could not contribute to the “signal” required to show thrombectomy was beneficial. These reviewers believed this had been the biggest problem with the IMS-3 study, even though this interpretation could be challenged by many alternate explanations for the inconclusive IMS-3 results [7].

In a care trial, the treatment of a patient who may not clinically require a test (for example, a patient with atrial fibrillation who presents with hemiplegia, aphasia, and normal CT except for a dense MCA) should not be delayed in order to comply with the tests required by the protocol. Not all the hospitals are organized or staffed to provide CTA with timely interpretation. As CTA was mandated in the guidelines for thrombectomy [31, 32], transfers are commonly delayed by an hour or more to fulfill this criterion, a constraint that is not in the interest of patients. Even at high-volume thrombectomy centers, addition of CTA delays time to groin puncture when compared with selection by hyperdense vessels on CT [33]. Worse, some trials have imposed delays on patients that would eventually not be included in the trial: the ESCAPE protocol, for example, required that “Stroke patients who have received i.v. tPA in a drip-and-ship paradigm must fulfill inclusion/exclusion criteria after *repeat* clinical *and imaging criteria* at the ESCAPE site”. [9] It is remarkable that none of the ethics committees that approved those studies recognized that this time-consuming and potentially deleterious protocol item was being forced upon non-participants. We emphasize that ethical trials must not impose per-protocol items to improve the efficiency of the trial at the potential cost of an adverse effect on patients.

Another hallmark of pragmatic and care trials is the value of showing improved outcomes in a diversity of patients and care settings that increases confidence that the results may truly apply in normal clinical practice. This is poorly understood, as illustrated by criticisms regarding selection according to the location of the occlusion and the severity of clinical presentations (as in the following reviewer’s quote): “‘The wisdom of mixing anterior circulation cases with basilar artery occlusion patients is highly questionable, since the outcomes are very different. Mortality is high, calling into question the judgment in case selection.” The truth is that trials always include patients that have “different outcomes”. It is unclear to us what could be the rationale for excluding patients with basilar occlusions; the same technique is used for both anterior and posterior circulation strokes; the carotid and middle cerebral arteries have perforators just like the basilar artery. All those patients required care, and the benefits of thrombectomy were yet to be proven, whether for basilar occlusions or those at other locations [19, 34].

Let us summarize the “reasons” and the scientific problems related to the selection of patients. One straightforward clinical reason for exclusion is when one of the treatments potentially being allocated could be harmful to a patient. But research-related reasons (“to decrease heterogeneity and maximize trial efficiency” and “to precisely characterize the population of patients to whom results will apply in the future”) do not apply in care trials. The reviewers were concerned that by not being selective enough, EASI might not have shown the benefits of therapy. As one reviewer wrote: “Such a care trial design could kill a therapy without giving it further chances for proving its worth.” Although such reasons may seem sound, the problem is: how is it possible to determine a priori (before the study is performed and the data collected) what the results of any particular subgroup will be? How is it possible to precisely aim at a target when no one knows where the target is? Certainly, patients always differ according to this or that characteristic, but we must differentiate patient heterogeneity (differences between patients) from heterogeneity of treatment effect (differences in treatment responses for different patients, which were at the time unknown). We must also remember that a clinician’s duty is to care for *all* patients, not only those who have good prognoses. Many trial protocols were deliberate attempts to show treatment in a good light. For example, the ESCAPE protocol stated: “We adopt a fast image acquisition (good scan, small core) occlusion model of patient selection and believe that this group of patients are the best ones to show a large magnitude of effect and provide the proof that endovascular therapy is the right treatment for patients with stroke”. But selecting patients to show a large treatment effect is scientifically and ethically problematic. The scientific problem concerns the impact of the selection of patients included in the study on the generalization of trial results to future patients. If the trial showed that thrombectomy can work in a narrow selection of best cases, it cannot support the eventual treatment of all other patients [35]. Unfortunately, once major trials have already claimed a large treatment effect for the core of the spectrum of patients, conducting a trial on the disparate remaining groups of patients (according to time, location, extent of infarction etc.) may become extremely difficult, the more so without the now-gone initial enthusiasm of the clinical community and the financial incentives of the Industry. When certain patients are excluded from trial participation by design through strict selection, similar future patients are at risk of being automatically excluded from future treatment as a result of new guidelines and indications following a positive trial. But no one knows whether these patients could also benefit from the treatment. We have witnessed this mistake in the IV tPA trials [36, 37]. Patients that could have benefited from intravenous thrombolysis have been denied effective therapy for decades because the selection criteria of some of the initial studies were too restrictive [36, 37]. Looking at the various thrombectomy trials, it would seem that the investigators’ desire to show therapy in a good light led them to overshoot the target, since even small prematurely interrupted trials showed overwhelming benefits. Clearly, we have missed patients that could have been helped by thrombectomy. But which patients? One study showed that the selection criteria of the various trials apply to a range that is as wide as 5–50% of patients with strokes [35]. Imagine the number of patients that would have been denied an effective treatment had we only conducted Extend-IA, for example, which concerned a minority of patients (5–10%) instead of the more inclusive MRCLEAN (40–50% of stroke patients). Now similar questions are raised for two positive thrombectomy trials for late presentation of patients with stroke in the 6–24 h window [28, 29], who were carefully selected, perhaps over selected by advanced imaging criteria using CT perfusion and magnetic resonance imaging (MRI). The results of these two trials were overwhelmingly positive, with a number needed to treat as low as 2. This implies that there are many other patients, with larger core infarct than that included in DEFUSE 3 and DAWN, that could benefit from thrombectomy in a late window. Sadly, the stroke guidelines now cite recommendation on using these advanced imaging criteria for thrombectomy in the late window [38].

If we are to question the merits of thrombectomy at the same time we offer thrombectomy to patients in need of care, why not question it for *all* patients who are potential candidates for the treatment? If some investigators believe that they can identify a subgroup of patients that are more likely to benefit, they can use those criteria to a priori define a subgroup for interim (and final) analyses, but that in and of itself is no reason to deny participation to all the other patients.

There are ethical problems with stringent selection of patients. They are related to the scientific concerns, but they are more immediate: What should we do with all other patients excluded from trial participation? Should we treat them with thrombectomy or standard care? This problem concerns the ethics of the clinicians’ participation. Here is the moral dilemma: physicians who propose randomized allocation only to those patients whom they believe are most likely to benefit, while continuing to perform thrombectomy in less favorable patients, put themselves in a morally precarious position; they cannot escape the fact that while their clinical duty is to treat each patient according to their best medical interest, participation in a trial that “sieves”, or selects highly favorable patients means that they are sacrificing selected patients for the sake of a research protocol, for knowledge, for monetary compensation, or for future patients. This is, to say the least, unfair if not contrary to medical ethics.

It seems as if the reviewers of the EASI manuscript, by asking for a more explanatory trial design, were implicitly endorsing the widely held notion for a clean-cut separation of research and practice [39]. According to this conception, research is foreign to medical care. It may be needed to show or prove that a treatment works, but it is a necessary evil, a brief interlude during which care ethics must be suspended for the sake of knowledge. Thus, some altruistic patients – the smallest number possible – are used to show as efficiently as possible that thrombectomy *can* work. The narrowly defined selection criteria, the rigid protocols, the blinded assessment of outcomes, the explanatory tests that are not necessary to urgent care, all serve to maximize the “yield” of the trial or to increase our understanding of how thrombectomy could help future patients [40]. Trials are at best justified by voluntary participation of individuals willing to contribute to advance medical knowledge. Recently, this has even been proposed as a moral obligation on patients [41].

Many unfortunate consequences follow from this conception: in a nutshell, the trials are not designed in the best medical interest of participants. This, in turn, justifies restrictive regulations that obstruct clinicians from testing their interventions. It also explains some of the reluctance patients and clinicians feel towards RCT participation, and why so many interventions have never been properly tested. Now, after the thrombectomy trials have been unequivocally positive, the guidelines that have subsequently been formulated show the error quite clearly: a long list of disparate, excluded patient remains for whom no one knows whether thrombectomy is indicated [38]. More inclusive trials would now have to be repeated. This would not have been the problem with a trial that was all-inclusive from the outset.

For decades we have been confronted with the opposition between what is alleged as sound trial design and good research conduct and the ethics of care. Care trials do not recognize this artificial research–care separation. For sure, there must be a separation, not between research and care, but between experimental (unvalidated) and standard (validated) care, because our innovations may turn out to be harmful. This is why care trials cannot be replaced by observational studies. Experimental care should not be prescribed as normal care. It should be provided as a 50% chance of receiving the innovation, balanced by a 50% chance of receiving standard care, because for the time being, no one knows which is best. Once the innovation is shown superior, it becomes validated care, which can then be prescribed. Optimal, outcome-based medical care (care in the best medical interest of the patient) is care previously shown to improve patient outcomes, when trial results are available, but a randomized care trial, when no such evidence is yet available. Without such norms, we cannot control for each patient the risks of entering unknown territory, and there is no way of demarcating known (validated) from unknown territories (unvalidated care). What has yet to be recognized is how specific care trial methods should be integrated into practice to be at the service of the care of current patients for whom no one knows what to do. Care trials have been designed to reconcile care and clinical research ethics, and offer optimal care in the presence of uncertainty, because it is impossible that good medical practice would be incompatible with the doctor verifying whether his practice does good or harm. On the other hand, care research protocols need to be adapted, because clinical research must not be designed in the same manner as laboratory research [2]. It is a science of practice, and it must respect the needs and interests of individual patients. Central to this idea are a number of important ethical concepts, including the obligation to provide optimal care for each patient, and the obligation to avoid imposing nonclinical risks and burdens [41]. Care trials are designed to be the way to offer care interventions that have yet to show they improve patient outcomes, in the best medical interest of current patients. The various criticisms that we have reviewed show that these principles are yet a long way from being recognized.

## Conclusion

Care trials such as EASI are at the pragmatic end of the explanatory–pragmatic spectrum of trial designs. Pragmatic design choices are commonly judged to be scientifically inferior to explanatory choices, but this only reveals a misplaced bias in favor of laboratory methods that are not appropriate to a science of clinical practice. Trial methods can be put at the service of patient care in the presence of uncertainty. A reconciliation of scientific methods with the central ethical principle of care is in order. More work is needed to integrate sound scientific methods and ethical care in the best medical interest of current patients.

## Abbreviations

CT: Computed tomography
CTA: Computed tomography angiography
EASI: Endovascular acute stroke intervention
IA: Intra-arterial
ICA: Internal carotid artery
IMS-3: Interventional Management of Stroke (IMS) III Trial
iv tPA: Intravenous tissue plasminogen activator
MCA: Middle cerebral artery
mRS: Modified Rankin Scale
NIHSS: National Institute of Health (NIH) Stroke Scale
PRECIS: Pragmatic-explanatory continuum indicator summary
RCT: Randomized controlled trial
tPA: Tissue Plasminogen Activator
